# Practice Characteristics of Board-certified Pediatric Anesthesiologists in the US: A Nationwide Survey

**DOI:** 10.7759/cureus.5745

**Published:** 2019-09-24

**Authors:** Matthew Muffly, David Scheinker, Tyler Muffly, Mark Singleton, Rita Agarwal, Anita Honkanen

**Affiliations:** 1 Anesthesiology, Stanford University, Stanford, USA; 2 Department of Management Science and Engineering, Stanford University, Stanford, USA; 3 Department of Obstetrics and Gynecology, Denver Health Hospital Authority, Denver, USA

**Keywords:** anesthesiology, pediatrics, clinical practice patterns, surveys and questionnaires

## Abstract

Introduction

We conducted a survey to describe the practice characteristics of anesthesiologists who have passed the American Board of Anesthesiology (ABA) Pediatric Anesthesiology Certification Examination.

Methods

In July 2017, a list of anesthesiologists who had taken the ABA Pediatric Anesthesiology Certification Examination (hereafter referred to as "pediatric anesthesiologists") was obtained from the American Board of Anesthesiologists (theaba.org). Email contact information for these individuals was collected from departmental rosters, email distribution lists, hospital or anesthesia group profiles, manuscript author contact information, website source code, and other publicly available online sources. The survey was designed using Qualtrics (Qualtrics, Provo, Utah; Seattle, Washington), a web-based tool, to ascertain residency/fellowship training history and current practice characteristics that includes: years in practice, clinical work hours per week, primary hospital setting, practice type, supervision model, estimated percentage of cases by patient age group, and percentage of respondents who cared for any patient undergoing a fellowship-level index cases within the previous year. The invitation to complete the survey included a financial incentive - the chance to win one of twenty $50 Amazon gift cards.

Results

There were 3,492 anesthesiologists who had taken the Pediatric Anesthesiology Certification Examination since 2013. Surveys were sent to those whom an email address was identified (2,681) and 962 complete survey responses were received (35.9%, 962/2,681). Over 80% (785) of respondents completed a pediatric anesthesiology fellowship. Of these, 485 respondents (50.4%) work in academic practice, 212 (22.0%) in private practice, 233 (24.2%) in private practice and have academic affiliations, and 32 (3.3%) as locum tenens or in other practice settings. The majority of respondents (64.3%) in academic practice work in freestanding children’s hospitals. Pediatric anesthesiologists in academic practice and private practice with academic affiliations reported caring for a greater number of younger children and doing a wider variety of index cases than respondents in private practice.

Conclusion

The extent to which pediatric anesthesiologists care for pediatric patients - particularly young children and those undergoing complex cases - varies. The variability in practice characteristics is likely a result of differences in hospital type, anesthesia practice type, geographic location, and other factors.

## Introduction

The field of pediatric anesthesiology has evolved and grown substantially since it became an American College for Graduate Medical Education (ACGME) approved fellowship in 1997 [1]. In 2011, the pediatric anesthesia fellowship entered the National Residency Matching Program (NRMP); the American Board of Anesthesiology (ABA) began offering the Pediatric Anesthesiology Certification Examination in 2013.

Until recently, no comprehensive list of pediatric anesthesiologists in the United States (US) existed. In 2015, our group created the first database of pediatric anesthesiologist demographic characteristics and geographic locations in the US [2]. Despite the improved understanding of pediatric anesthesiologist geographic location and demographics, the practice characteristics of pediatric anesthesiologists in the US remain largely unknown.

Understanding practice characteristics of pediatric anesthesiologists - the anesthesia practice types and hospital settings in which they work, categories of patients they care for, and for what types of procedures - are necessary for a better understanding of the spectrum of anesthesia care provided to pediatric patients in the US and is relevant for workforce planning, fellowship education, and outcome research. Therefore, we conducted a survey of the practice characteristics of anesthesiologists who passed the ABA Pediatric Anesthesiology Certification Examination. 

## Materials and methods

In July 2017, a list of anesthesiologists who registered for the ABA Pediatric Anesthesiology Certification Examination was obtained at "theaba.org" using the "Advanced Search" function. From October to December 2017, email contact information for these individuals was collected from departmental rosters, email distribution lists, hospital or anesthesia group profiles, manuscript author contact information, website source code, and other publicly available online sources. 

The Stanford University Administrative Panel on Human Subjects in Medical Research waived the requirement for written informed consent and approved the study.

Survey design

The survey instrument was designed and distributed using Qualtrics (Qualtrics, Provo, Utah; Seattle, Washington), a web-based tool for creating and conducting online surveys. Survey questions were designed to determine residency/fellowship training history and current practice characteristics including years in practice, clinical work hours per week, primary hospital setting, practice type, supervision model, estimated percentage of cases by patient age group, and percentage of respondents who cared for any patient undergoing a fellowship-level index cases within the previous year. Fellowship-level index cases are an indicator of complex pediatric anesthesia care and include airway surgery (excluding tonsillectomy and adenoidectomy), cardiac surgery with cardiopulmonary bypass, cardiac surgery without cardiopulmonary bypass, craniofacial surgery, intra-abdominal/intracavitary surgery, intracranial neurosurgery, intrathoracic non-cardiac surgery, major orthopaedic surgery, neonatal emergency surgery, and solid organ transplant [3].

Anesthesiologist sex was imported from an existing database. The years in practice was estimated based on the time of primary board certification in anesthesia until the survey date. Survey responses were retained for those who fully completed the demographics and practice characteristics sections of the survey. Respondents and non-respondents who registered for the ABA Pediatric Anesthesiology Certification Examination but did not subsequently pass were excluded from the analysis as to include only anesthesiologists with ABA Pediatric Anesthesiology Certification.

The survey was first distributed to 20 members of the authors’ department and revised based on their suggestions for clarity and for improvement. In January 2018, the revised survey was emailed to all pediatric anesthesiologists whose email address had been identified (Supplement 1). The invitation to complete the survey included a financial incentive - the chance to win one of twenty $50 Amazon gift cards.

Two reminders were sent to survey non-respondents within the next 30 days. The survey was closed six weeks after the initial email.

## Results

**Figure 1:**
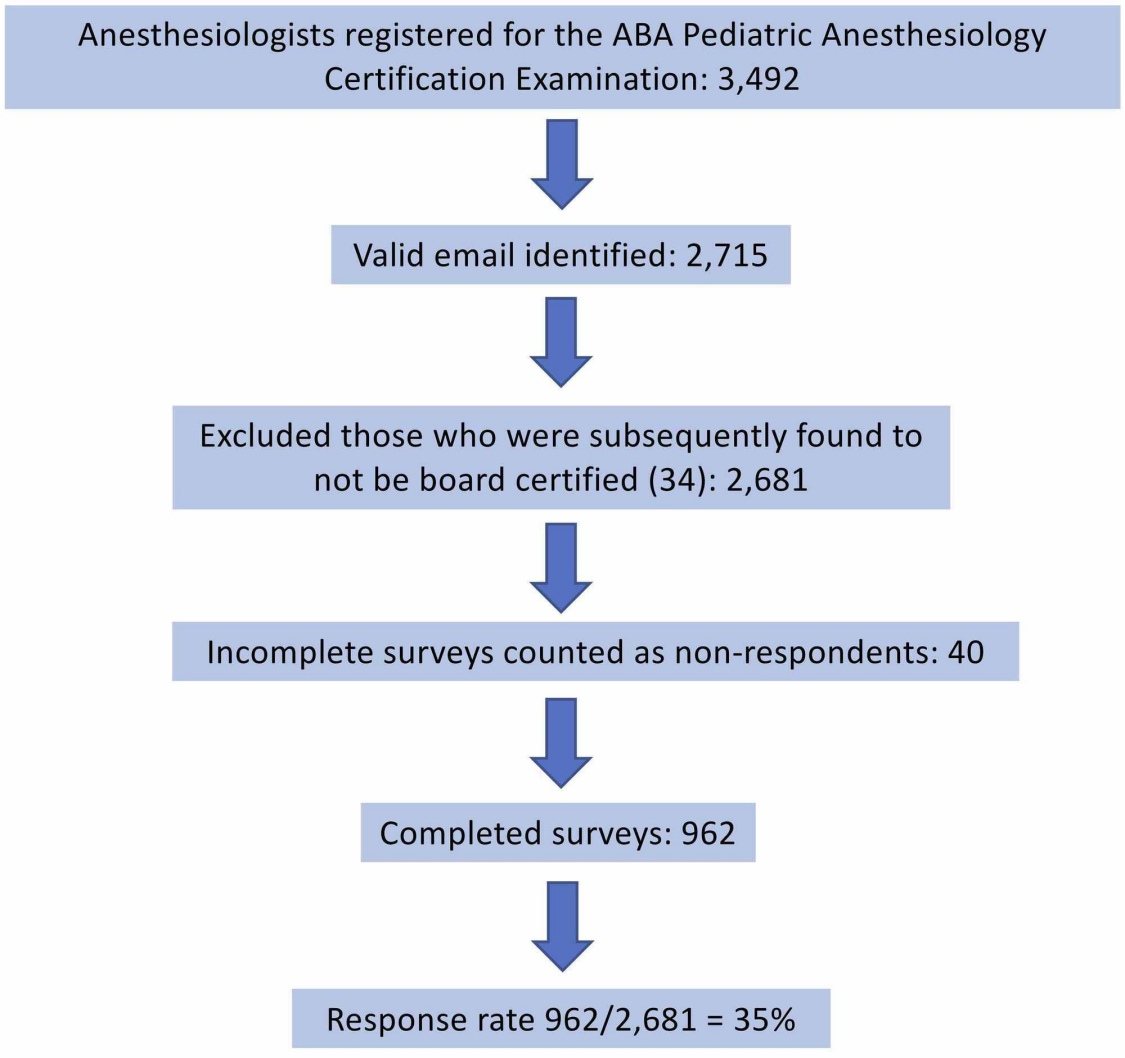
Flow Diagram of Study Participants

There were 3,492 anesthesiologists who had taken or were registered for the ABA Pediatric Anesthesiology Certification Examination in July, 2017. Surveys were sent to 2,681 individuals whose emails were identified; 962 (35.9% of those emailed) complete survey responses were received (Figure 1).

Years in practice, sex, geographic location, and training characteristics are reported in Table 1. Over 80% (785) of respondents completed a pediatric anesthesiology fellowship. Sixty-nine respondents (7%) reported completing an advanced second-year fellowship, mostly in pediatric cardiac anesthesia.

**Table 1:** Demographic and Practice Characteristics of Respondents and Non-respondents

	Respondents	Non-Respondents
	962 (35.9)	1719 (64.1)
Years in practice, median (IQR)	10 (5 - 20)	9 (5 - 18)
Sex		
Female	446 (46.4)	781 (45.4)
Male	516 (53.6)	938 (54.6)
US Census Division (States)		
East North Central (IL, IN, MI, OH, WI)	151 15.7)	289 (16.8)
East South Central (AL, KY, MS, TN)	30 (3.1)	62 (3.6)
Middle Atlantic (NJ, NY, PA)	146 (15.2)	276 (16.1)
Mountain (AZ, CO, ID, MT, NM, NV, UT, WY)	60 (6.2)	114 (6.6)
New England (CT, MA, ME, NH, RI, VT)	74 (7.7)	113 (6.6)
Pacific (AK, CA, HI, OR, WA)	171 (17.8)	254 (14.8)
South Atlantic (DE, DC, FL, GA, MD, NC, SC, VA, WV)	163 (16.9)	283 (16.5)
West North Central (IA, KS, MN, MO, ND, NE, SD)	65 (6.8)	115 (6.7)
West South Central (AR, LA, OK, TX)	100 (10.4)	212 (12.3)
Puerto Rico	2 (0.2)	1 (0.1)
Completed additional residency		
No	804 (83.6)	
Yes	158 (16.4)	
Pediatrics	140 (88.6)	
Internal medicine	10 (6.3)	
Family medicine	3 (1.9)	
Surgery	3 (1.9)	
OB/Gyn	1 (0.6)	
Emergency	1 (0.6)	
Completed pediatric anesthesiology fellowship		
No	177 (18.4)	
Yes	785 (81.6)	
Completed advanced second-year fellowship		
No	716 (74.4)	
Yes	69 (7.2)	
Cardiac	36 (52.2)	
Research	10 (14.5)	
Pain	6 (8.7)	
Regional	6 (8.7)	
QI/Patient safety	2 (2.9)	
Education	2 (2.9)	
Other	7 (10.1)	
Did not specify	177 (18.4)	
Practice type		
Academic	485 (50.4)	
Private practice	212 (22.0)	
Private practice with academic affiliation	233 (24.2)	
Locum tenens/other	32 (3.3)	

Of the 962 respondents, 485 (50.4%) work in an academic practice, 212 (22.0%) in private practice, 233 (24.2%) in private practice and have academic affiliations, and 32 (3.3%) as locum tenens or in some other practice setting.

Pediatric anesthesiologists in academic practice reported working fewer clinical hours than those in private practice or in a private practice with an academic affiliation. The majority of pediatric anesthesiologists (64%) in academic practice work in a freestanding children’s hospital at least 50% of the time, whereas only 17% of those in private practice, and 47% of those in private practice with an academic affiliation do the same. The majority of anesthesiologists in academic practice supervise residents (96.7%), fellows (80.8%), certified registered nurse anesthetists (CRNAs; 86.6%), and non-anesthesia trainees (58.6%) whereas the majority in private practice supervise CRNAs only (Table 2). More than 20% of anesthesiologists in academic practice supervise certified anesthesiology assistants. 

**Table 2:** Practice Characteristics by Practice Type

	Overall (n=962)	Academic Practice (n=485)	Private Practice (n=212)	Private Practice and an Academic Affiliation (n=233)	Locum Tenens/Other (n=32)
Years in practice, median (IQR)		10 (5 - 20)	8 (4 - 19)	11 (5 - 21)	12.5 (5.75 - 19.25)
Sex, n (%)					
Female	446 (46.4)	255 (52.6)	95 (44.8)	80 (34.3)	16 (50.0)
Male	516 (53.6)	230 (47.4)	117 (55.2)	153 (65.6)	16 (50.0)
Clinical work hours per week, mean (SD)	46.9 (12.4)	44.9 (12.6)	48.5 (11.6)	50.3 (10.8)	42.4 (17.3)
Clinical work hours per week by sex, mean (SD)					
Female	45.0 (12.0)	44.0 (12.3)	46.2 (11.9)	47.4 (10.9)	43.4 (10.2)
Male	48.5 (12.5)	45.8 (12.9)	50.4 (11.0)	51.8 (10.4)	41.4 (22.0)
Hospital setting (at least 50% of time), n (%)^					
General hospital WITHOUT dedicated children’s unit	99 (10.3)	27 (5.6)	46 (21.7)	19 (8.2)	7 (21.9)
General hospital WITH dedicated children’s unit	343 (35.7)	144 (29.7)	98 (46.2)	91 (39.1)	10 (31.3)
Freestanding children’s hospital	470 (48.9)	312 (64.3)	36 (17.0)	110 (47.2)	12 (37.5)
Ambulatory surgery center	52 (5.4)	9 (1.9)	32 (15.1)	9 (3.9)	2 (6.3)
Other (VA, office-based, dental setting)	9 (0.9)	2 (0.4)	4 (1.9)	0 (0)	3 (9.4)
Respondents who supervise, n (%)					
Residents	648 (67.4)	469 (96.7)	19 (9.0)	152 (65.2)	8 (25.0)
Fellows	455 (47.3)	392 (80.8)	5 (2.4)	57 (24.5)	1 (3.1)
Certified registered nurse anesthetists	735 (76.4)	420 (86.6)	125 (59.0)	174 (74.7)	16 (50.0)
Certified anesthesiology assistants	160 (16.6)	106 (21.9)	18 (8.5)	31 (13.3)	5 (15.6)
Non-anesthesia trainees	434 (45.1)	284 (58.6)	45 (21.2)	100 (42.9)	5 (15.6)
Mean percentage of time by practice setting					
Operating room	92.7	90.7	95.5	95.1	87.5
Acute pain service	3.3	3.9	2.5	2.8	2
Chronic pain service	1.2	2.2	0.2	0.4	0
Pediatric intensive care	1.6	2.3	0.6	1	0.8
Other	1.2	0.9	1.1	0.7	9.7
Percentage of cases by age					
Infants/children (aged 0–2 years)	21.6	25.3	14.6	20.7	20.2
Children (aged three years to five years)	24.7	27.4	18.6	24.6	25.4
Older children (aged six years to 17 years)	24.8	28.2	16.9	25.4	23.1
Adults (aged 18+ years)	29	19.1	50	29.3	31.2
Number of anesthesiologists who cared for patients undergoing index cases within the past 12 months, n (%)					
Airway	814 (84.6)	427 (88.0)	162 (76.4)	205 (88.0)	20 (62.5)
Cardiac with CPB	192 (20.0)	11 (2.3)	25 (11.8)	54 (23.2)	1 (3.1)
Cardiac without CPB	263 (27.3)	151 (31.1)	37 (17.5)	72 (30.9)	3 (9.4)
Craniofacial	596 (62.0)	312 (64.3)	105 (49.5)	163 (70.0)	16 (50.0)
Intra-abdominal/intracavitary	840 (87.3)	438 (90.3)	171 (80.7)	210 (90.1)	21 (65.6)
Intracranial neurosurgery	651 (67.7)	362 (74.6)	99 (46.7)	175 (75.1)	15 (46.9)
Intrathoracic non-cardiac	679 (70.6)	373 (76.9)	119 (56.1)	172 (73.8)	15 (46.9)
Major orthopedic	732 (76.1)	386 (79.6)	134 (63.2)	190 (81.6)	22 (68.8)
Neonatal emergency	753 (78.3)	400 (82.5)	142 (67.0)	193 (82.8)	18 (56.3)
Solid organ transplant	300 (31.2)	222 (45.8)	10 (4.7)	63 (27.0)	5 (15.6)
ASA physical status greater than/equal to four	748 (77.8)	421 (86.8)	128 (60.4)	181 (77.7)	18 (56.3)
*Due to rounding, percentages may not sum to 100%					
^sum of percentages >100 because respondents who indicated 50% time were counted twice					

Pediatric anesthesiologists in academic practice indicated that the majority of their patients were five years old or younger (52.7%) versus 33.2% for those in private practice, and 45.3% for those in private practice with an academic affiliation. All practice characteristics, stratified by anesthesia practice type, are shown in Table 2.

## Discussion

The results of this survey illustrate the practice characteristics of anesthesiologists who successfully completed the ABA Pediatric Anesthesia Certification. An understanding of their practice characteristics is important for workforce planning, fellowship education, outcome research, and optimizing the care of children.

The results of this survey suggest that, in general, respondents in academic practice or private practice with academic affiliations care for a higher percentage of younger patients as well for as patients undergoing a wider variety of fellow-level index cases, than those in private practice. Over 90% of all respondents, regardless of practice type, reported caring for patients undergoing at least one index-level case type in the past year. On the other hand, nearly one in 10 respondents in private practice (or one in 20 respondents in academic practice or academic practice with an academic affiliation) reported caring for no patients undergoing an index case within the preceding year. 

This issue is particularly timely, given the potential effect of the American College of Surgeon's Children's Surgery Verification Quality Improvement Program on the distribution of pediatric surgery in the US. It is possible that with regionalization of pediatric surgical procedures, the number of pediatric cases per pediatric anesthesiologist at non-tertiary care children's hospitals will decrease. This raises questions about how anesthesiologists will maintain clinical competency given a potential reduction in the number of children undergoing care in the community hospital setting.

There are certain limitations that should be considered when interpreting the results of this survey. The survey describes the practice characteristics of anesthesiologists who passed the ABA Pediatric Anesthesiology Certification Examination; the respondents may be biased toward the academic end of the practice spectrum, where certification is often required. In addition, there are many anesthesiologists who did not take the ABA Pediatric Anesthesiology Certification Examination who nevertheless care for children routinely. Anesthesiologists who trained before the advent of time-limited anesthesiology subspecialty boards hold board certifications that include all subspecialties. The survey misses a large number of legitimately qualified practitioners who hold "board certified" status in the field of anesthesiology - inclusive of pediatric anesthesiology. In addition, the survey response rate was low (35.9%), thus raising the possibility of bias in the results. Although respondents and non-respondents appeared similar in terms of sex, years in practice, and geographic location, they may differ in unobservable ways, thus potentially limiting the generalizability of the results. Nevertheless, this survey gives us an improved understanding of the practice characteristics of those pediatric anesthesiologists with ABA Pediatric Anesthesiology Certification.

## Conclusions

The practice characteristics of pediatric anesthesiologists are now better understood. The extent to which pediatric anesthesiologists care for pediatric patients - particularly young children and those undergoing complex cases - varies. The variability in practice characteristics is likely a result of differences in hospital type, anesthesia practice type, geographic location, and other factors. 

## Appendices

Practice Characteristics of Board-Certified Pediatric Anesthesiologists

Do you agree to proceed?

o Yes  (1)

o No  (2)

The first set of questions is about your TRAINING. 

1. Did you complete a residency IN ADDITION to Anesthesiology?

o Yes  (1)

o No  (2)

Which additional residency did you complete?

▼ _______________________________________

2. Did you complete a Pediatric Anesthesiology Fellowship?

o Yes  (1)

o No  (2)

3. When did you complete your Pediatric Anesthesiology Fellowship?

▼ 2017 (1) ... 1967 (51)

4. After Pediatric Anesthesiology Fellowship, did you complete an Advanced Second Year Fellowship in Pediatric Anesthesiology?

o Yes  (1)

o No  (2)

What was your Advanced Second Year Fellowship in Pediatric Anesthesiology (select all that apply)?

▢        Cardiac  (1)

▢        Pain  (2)

▢        Regional  (3)

▢        Research  (4)

▢        Quality Improvement/Patient Safety  (5)

▢        Education  (8)

▢        Other  (6) ________________________________________________

5. Which of the following best describes your current anesthesiology practice?

o Academic Practice  (3)

o Private Practice  (1)

o Private Practice with Academic Affiliation  (2)

o Locum Tenens  (5)

o Other, please describe:  (4) ________________________________________________

6. Please indicate if you currently supervise any of the following (select all that apply):

▢        Anesthesiology Residents  (1)

▢        Anesthesiology Fellows  (2)

▢        Certified Registered Nurse Anesthetists (CRNA)  (3)

▢        Anesthesia Assistants (AA)  (6)

▢        Non-anesthesia Trainees  (4)

7. What percentage of time do you care for patients in the following settings? (must total 100%)

General Adult Hospital WITHOUT dedicated areas for care of children : _______  (1)

General Adult Hospital WITH dedicated areas for care of children : _______  (2)

Freestanding Children's Hospital : _______  (3)

Ambulatory Surgery Center : _______  (4)

Veteran's Administration : _______  (6)

Office-Based Practice : _______  (5)

Dental Practice : _______  (7)

Other : _______  (8)

Total : ________

8. On average, how many hours per week do you spend directly caring for patients?

___

9. What is the ZIP code of your main employment site?

___

10. What percentage of time do you care for patients in each of the following services/settings? (must total 100%)

Operating Room and Out-of-Operating Room Anesthetizing Locations : _______  

Acute Pain/Regional Service  : _______  

Chronic Pain Service  : _______  

Pediatric Intensive Care Unit  : _______  

Other  : _______  

Total : ________

11. Estimate the percentage of patients in each age cohort you care for in your practice (must total 100%) 
 

Age 0 -  2 Years : _______  

Age 3 - 5 Years : _______  

Age 6 - 17 Years : _______  

Age 18+ Years : _______  

Total : ________

12. Indicate the types of pediatric cases (age 0-17 years) that you have done in the past 12 months (select all that apply)

▢        Airway (not including tonsillectomy and adenoidectomy)  (1)

▢        Cardiac‐with bypass   (2)

▢        Cardiac‐without bypass   (3)

▢        Craniofacial (not including cleft lips/palates)  (4)

▢        Intra‐abdominal/intracavitary   (5)

▢        Intracranial neurosurgery   (6)

▢        Intrathoracic non‐cardiac   (7)

▢        Major orthopedic  (8)

▢        Neonatal emergency   (9)

▢        Solid organ transplant   (10)

▢        ASA Physical Status Greater than or Equal to 4  (11)

13. How satisfied are you with your current practice?    

Likert Scale: Very Dissatisfied (0) - Very Satisfied (10)

Reasons for degree of satisfaction (optional):

________________________________________________________________

14. Is there a need for additional pediatric anesthesiologists in your geographic area?

o Yes  

o No  

o Not Sure  

15. Is there any additional information you would like to add about the job market in your area (optional)?

________________________________________________________________

Thank you, you are done! Would you like to enter your name into the drawing for one of twenty $50 Amazon Gift Cards?

o Yes  (1)

o No  (2)

Name:

________________________________________________________________

Email:

________________________________________________________________
